# Monomicrobial necrotizing soft tissue infection of upper extremity caused by *Eggerthia catenaformis*

**DOI:** 10.1093/jscr/rjaf769

**Published:** 2025-10-03

**Authors:** Nadeem Chaudhry, Abid Qureshi, Dhipthika Srinivasan, Michael Davrayev, Natasha Vo, Heidi Wallour, Minnah Chaudhry

**Affiliations:** Department of Plastic Surgery, The Brooklyn Hospital Center, Icahn School of Medicine, 121 DeKalb Ave, Brooklyn NY 11201, United States; Department of Surgery, The Brooklyn Hospital Center, 121 DeKalb Ave, Brooklyn NY 11201, United States; Department of Surgery, The Brooklyn Hospital Center, 121 DeKalb Ave, Brooklyn NY 11201, United States; St. George’s University School of Medicine, University Centre Grenada, West Indies, Grenada; St. George’s University School of Medicine, University Centre Grenada, West Indies, Grenada; St. George’s University School of Medicine, University Centre Grenada, West Indies, Grenada; Department of Surgery, The Brooklyn Hospital Center, 121 DeKalb Ave, Brooklyn NY 11201, United States

**Keywords:** necrotizing infection, soft tissue, *Eggerthia catenaformis*

## Abstract

Necrotizing soft tissue infections are characterized by fulminant tissue destruction that are rapidly progressive in nature, occurring as a result of trauma, minor skin lesions, nonpenetrating injuries, as well as in post-surgical and immunocompromised patients. *Eggerthia catenaformis* has mostly been reported linked to dental abscesses but this report presents a case of a poor controlled diabetic with *E. catenaformis* induced necrotizing infection. Forty nine-year-old male presented to the emergency department with left arm pain and swelling, associated with foul smelling drainage. Physical examination exhibited left upper extremity tenderness, erythema, fluctuance, and crepitus. Patient was taken to the operating room for forearm fasciotomy, amputation of third digit, and washout. Cultures were positive for *E. catenaformis*. The pathogenicity and severity of *E. catenaformis* is mostly unknown. Clinicians should be aware of this rare entity for its complexity and management worldwide.

## Introduction

Necrotizing soft tissue infections (NSTIs) are characterized by fulminant tissue destruction that are severe and rapidly progressive in nature, occurring as a result of trauma, minor skin lesions, nonpenetrating injuries, as well as in post-surgical and immunocompromised patients [1]. NSTIs commonly affect the skin, subcutaneous adipose tissue, fascia, and muscle. Arising from breaks in the epithelial or mucosal lining, NSTIs can be further classified by anatomical location, infection depth, and microbiology. There are four classifications of NSTIs: Type 1 is polymicrobial, type 2 is monomicrobial commonly caused by group A Streptococcus, Type 3 involves either *Clostridium* or organisms associated with freshwater exposure, and Type 4 is seen in immunocompromised patients with fungal infections [2]. *Eggerthia catenaformis* is a Gram-positive, non-spore-forming, rod-shaped anaerobic bacteria that is part of the flora in the gastrointestinal tract [3, 4]. *Eggerthia catenaformis* has mostly been reported in prior literature linked to dental abscesses. This report presents a case of a patient with poor controlled diabetes mellitus with *E. catenaformis* induced NSTI.

## Case presentation

**Figure 1:**
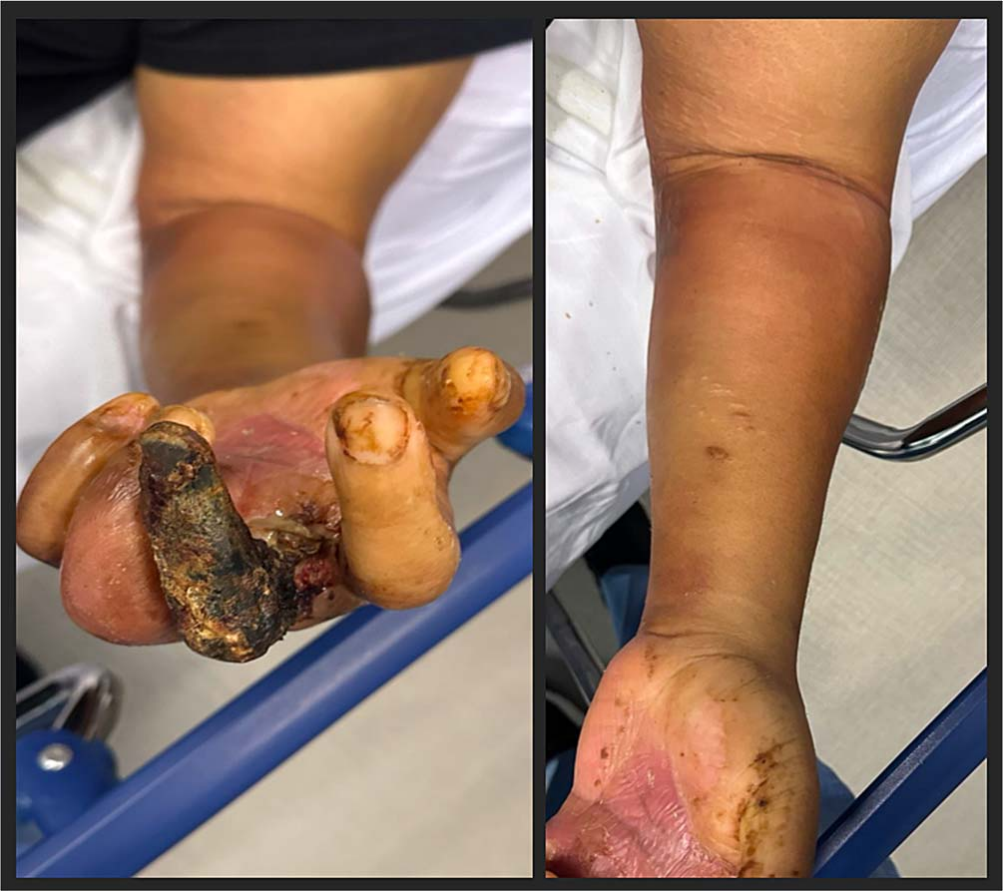
Initial presentation of left upper extremity. Third digit with gangrenous necrosis, with erythema and underlying crepitus of the arm (left picture). Forearm demonstrating erythema with underlying crepitus (right picture).

**Figure 2:**
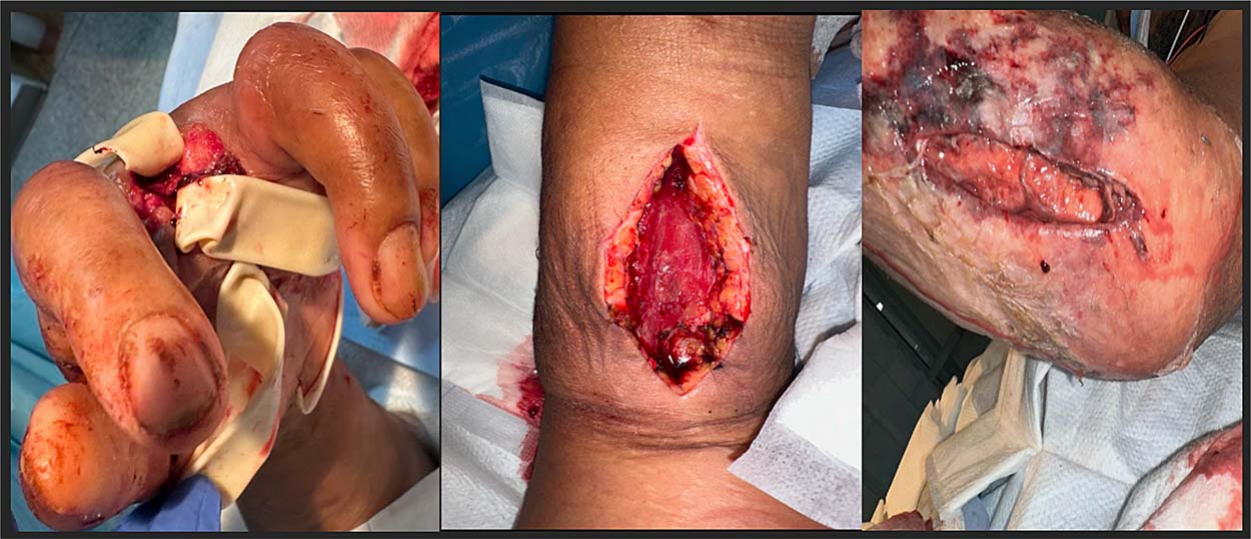
Postoperative after left upper extremity amputation and fasciotomy. Left third digit with penrose drain placement (left). Fasciotomy of volar aspect of forearm (middle). Fasciotomy of the dorsal aspect of the forearm (right).

A 49-year-old male presented to the emergency department with left arm pain and swelling, associated with foul smelling drainage. He reported the symptoms initially were waxing and waning over the last month, but over the last week progressively worsened. Patient reported continuously biting his fingers and hand, with progressive darkening of the third and fourth left upper extremity digits over that time span. However, peripheral radial and ulnar pulses were intact. Past medical history was significant for type 2 diabetes mellitus, which was poorly controlled due to non-compliance of medications. Physical examination exhibited left upper extremity tenderness, erythema, fluctuance, and crepitus extending from elbow region distally to the hand; there was active purulent drainage and third digit was completely necrotic and gangrenous with small aspect of bone exposed (Fig. 1). Laboratory workup showed leukocytosis (WBC 13.6) with a left neutrophil shift (83%). Computer tomography (CT) of left upper extremity showed extensive subcutaneous emphysema throughout the left forearm soft tissues extending into and overlying the second and third digits, consistent with necrotizing infection. Upon discussion with the patient, he was only amenable for incision and drainage of the infected regions of the forearm and amputation of the necrotic digit. Patient was taken to the operating room for left forearm fasciotomy of both volar and dorsal compartments, carpal tunnel fasciotomy, amputation of third digit, washout, with penrose drain placement (Fig. 2). Patient was upgraded to the surgical intensive care unit for postoperative care. Intraoperative cultures taken grew *E. catenaformis* solely. On Postoperative day 1, the patient was noted to have copious amounts of pus coming out of the dorsal forearm incision prompting a take back to the operating room. Patient underwent washout of the prior incision sites with debridement of any non-viable tissue. Patient was kept on intravenous antibiotic therapy with Meropenem for 4 weeks and required daily wound care till he was stable for a subacute rehabilitation facility. Patient was discharged with follow-ups for surgery and infectious disease clinics, however, was lost to follow-up.

## Discussion

NSTI is a broad term for multiple subsets of life-threatening soft tissue infections, affecting one or multiple layers of the skin from superficial to deeper structures, such as underlying soft tissue. Subcategories of this infection are described based on the anatomical location, depth of infection, and associated organism. In the United States, there are about 1000 cases per year [2]. Worldwide incidence ranges from 0.3–5 cases per 100 000 in Europe and up to 15.5 cases per 100 000 in Asia, with reported mortality rates from 20% to 32% [5]. The classification of NSTI is categorized into four classes. Type I is polymicrobial and is the most common type, accounting for up to 90% of the cases. Type II is monomicrobial and most commonly associated with beta-hemolytic *Streptococcus* A (*Streptococcus pyogenes*) but various other bacteria are linked as well, including *E. catenaformis*. *Aeromonas,* and *Vibrio* species, associated with seawater exposure along with *Clostridium*, fall under Type III. Lastly, Type IV is associated with fungal organisms, such as *Candida* and zygomycetes [6].

The pathophysiology of necrotizing soft tissue infection in all types involves destruction of skin and soft tissue below it is accomplished through several mechanisms. NSTI can occur from a break in the skin secondary to trauma or surgery. Different pathogens can enter and spread along the skin layers and underlying tissue. As it does so, it activates inflammatory response, local thrombosis, and subsequent ischemia and necrosis at any level it reaches. This vicious cycle can occur and is a common mechanism for all the potential causal pathogens [7]. Type 1 is highly associated with elderly patients with comorbid conditions, with the presence of aerobic and anaerobic organisms causing further damage hemodynamically. Type II NSTI, if caused by *S. pyogenes*, causes tissue destruction via exotoxins, which often initiate a complex cascade of immune-related responses, including upregulation of cytotoxic T-cells and cytokine release, potentially leading to toxic shock syndrome. Type III NSTI, most commonly associated with *Clostridium* species, causes infection via alpha-toxins that are released into the muscle, causing degradation and, subsequently, necrosis [8].

*Eggerthia catenaformis*, initially found in the human gastrointestinal tract microbiota, was first isolated in human feces in 1953. It is a Gram-positive, non-spore forming anaerobic bacteria in the shape of irregular rods forming chains. Samples of *E. catenaformis* is a communal flora that has been cultured from human saliva and feces in healthy patients [4]. When it becomes pathogenic, *E. catenaformis* has mostly been known to cause dental abscesses, spreading hematogenously. However, there have been cases where *E. catenaformis* was present without dental abscesses [9]. There are several cases in which this organism causes overall systemic infection. Most recently published by Yang *et al.* was a case of NSTI found within the labia, which had extended into the left inguinal region [9]. There have been only a few case reports published about *E. catenaformis*, one for example causing NSTI in the abdominal wall and groin [10].

NSTI is mainly a clinical diagnosis along with a thorough history it can properly be identified and controlled expeditiously. Early recognition of signs and symptoms are vital. Most patients present with pain out of proportion, erythema, and swelling in some instances bullae and crepitus may be present. The use of imaging can be utilized to visualize the extent of the disease. Commonly done with X-ray but more useful are the utilization of CT and MRI imaging, remarkable for soft tissue swelling and subcutaneous air [11]. The cornerstone of management of NSTI is to aggressively stop the underlying infection and prevent further spread. The combination of medical management with initiating broad spectrum antibiotics with a surgical approach is crucial for treating these infections. Aggressive surgical debridement is important to explore the wound but further exploration after 24–48 h may be necessary to determine tissue viability and appropriate wound care postoperatively [12].

*Eggerthia catenaformis*, in its rarity, can cause various systemic infections, most commonly dental abscesses [13]. Excluding this report, there have seldom been a few case reports of NSTI caused by *E. catenaformis*. All of those cases had diabetes mellitus as a contributory co-morbidity. In this case, our patient also had poorly controlled diabetes, with extreme non-compliance with medications, presenting with a month-long symptomology. The pathogenicity and severity of *E. catenaformis* is mostly unknown. Clinicians should be aware of this rare entity and should be reported to determine its complexity and management worldwide.
